# Interactive Multimedia Reporting Technical Considerations: HIMSS-SIIM Collaborative White Paper

**DOI:** 10.1007/s10278-022-00658-z

**Published:** 2022-08-12

**Authors:** Seth J. Berkowitz, David Kwan, Toby C. Cornish, Elliot L. Silver, Karen S. Thullner, Alex Aisen, Marilyn M. Bui, Shawn D. Clark, David A. Clunie, Monief Eid, Douglas J. Hartman, Kinson Ho, Andrei Leontiev, Damien M. Luviano, Peter E. O’Toole, Anil V. Parwani, Nielsen S. Pereira, Veronica Rotemberg, David J. Vining, Cree M. Gaskin, Christopher J. Roth, Les R. Folio

**Affiliations:** 1 Department of Radiology, Beth Israel Deaconess Medical Center, Boston, MA USA; 2 Insygnia Consulting Inc, Toronto, ON Canada; 3 Department of Pathology, University of Colorado School of Medicine, Aurora, CO USA; 4 Argentix Informatics, Inc, Vancouver, BC Canada; 5 PenRad Technologies, Inc, Buffalo, MN USA; 6 Royal Philips, Raanana, Israel; 7 Department of Pathology, Moffitt Cancer Center and Research Institute, Tampa, FL USA; 8 University of Miami Hospitals and Clinics, Miami, FL USA; 9 PixelMed Publishing, LLC, Bangor, PA USA; 10 eHealth & Digital Transformation Agency, Ministry of Health, Riyadh, Saudi Arabia; 11 Department of Pathology, University of Pittsburgh Medical Center, Pittsburgh, PA USA; 12 Arterys, San Francisco, CA USA; 13 Visage Imaging, Inc, San Diego, CA USA; 14 Department of Surgery, Virginia Tech Carilion School of Medicine, Roanoke, VA USA; 15 mTuitive, Inc, Centerville, MA USA; 16 Department of Pathology, The Ohio State University, Columbus, OH USA; 17 OMF Radiology, Independent, Rio de Janeiro, RJ Brazil; 18 Dermatology Service, Memorial Sloan Kettering Cancer Center, New York, NY USA; 19 Department of Abdominal Imaging, MD Anderson Cancer Center, Houston, TX USA; 20 Department of Radiology and Medical Imaging, University of Virginia, Charlottesville, VA USA; 21 Department of Radiology, Duke University, Durham, NC USA; 22 Moffitt Cancer Center, Tampa, FL USA

**Keywords:** Enterprise imaging, Multimedia, Reporting, Interoperability, IT standards, Integration standards

## Abstract

Despite technological advances in the analysis of digital images for medical consultations, many health information systems lack the ability to correlate textual descriptions of image findings linked to the actual images. Images and reports often reside in separate silos in the medical record throughout the process of image viewing, report authoring, and report consumption. Forward-thinking centers and early adopters have created interactive reports with multimedia elements and embedded hyperlinks in reports that connect the narrative text with the related source images and measurements. Most of these solutions rely on proprietary single-vendor systems for viewing and reporting in the absence of any encompassing industry standards to facilitate interoperability with the electronic health record (EHR) and other systems. International standards have enabled the digitization of image acquisition, storage, viewing, and structured reporting. These provide the foundation to discuss enhanced reporting. Lessons learned in the digital transformation of radiology and pathology can serve as a basis for interactive multimedia reporting (IMR) across image-centric medical specialties. This paper describes the standard-based infrastructure and communications to fulfill recently defined clinical requirements through a consensus from an international workgroup of multidisciplinary medical specialists, informaticists, and industry participants. These efforts have led toward the development of an Integrating the Healthcare Enterprise (IHE) profile that will serve as a foundation for interoperable interactive multimedia reporting.

## Introduction

Many medical specialists review images while diagnosing and treating diseases. Diagnostic radiology and pathology are two prototypical image-centric specialties. Many authors [1, 2] have documented the somewhat primitive means through which these image-centric specialists document their findings:

“Despite the clear importance of the imaging report and despite radiologists’ daily work in a field defined by rapid progression of new techniques and innovative digital tools, most radiologists continue to create reports in a manner strikingly similar to that of their predecessors practicing 100 years ago” [3].

At a conceptual level, reporting workflow is similar for most image-centric specialists. An image is evaluated on a computer screen, video monitor, or microscope. Image-centric specialists create a report of their findings by either typing a narrative description of findings, dictating a report for subsequent transcription, or using a voice recognition application. The report is transmitted to the electronic health record (EHR), where the report guides patient management. In some specialties, the text report may be the only available documentation of the image findings. However, in other specialties, the source images are increasingly available for review. A surgeon may wish to review images of computed tomography (CT) scan for surgical planning. An oncologist may review a positron emission tomography (PET) scan to show a patient how the disease burden has changed. A primary care doctor may review a photograph taken by a dermatologist to assess the change in a skin lesion. Pathologists often show histologic images of biopsy or resection specimens to colleagues during multidisciplinary conferences/tumor boards.

The process of correlating the findings described in a text report to the findings in the source images requires expertise and effort on the part of the reader. The reader may misunderstand the meaning of a text report without knowledge of image interpretation. Although technical systems for the digital creation, storage, and viewing of images and reports have matured, image and report data streams often remain segregated in the medical record throughout their lifecycle. There is rarely a direct connection between images, measurements, annotations, and key images with the description, identification, and image references contained in a narrative report.

**Fig. 1:**
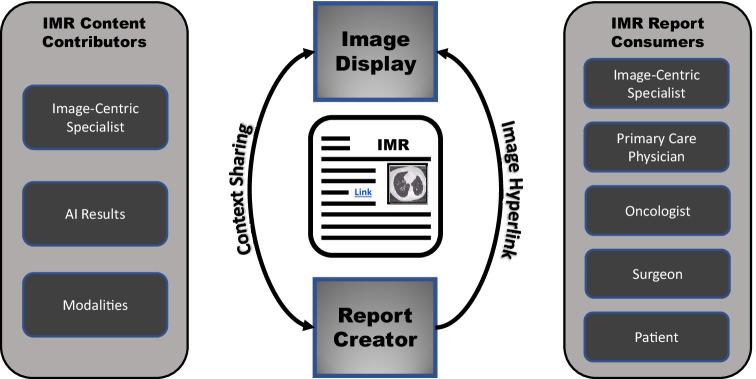
Graphic representation of how IMR enables the communication between content contributors (image-centric specialists) and information consumers (primary care providers, patients, other specialists)

**Fig. 2:**
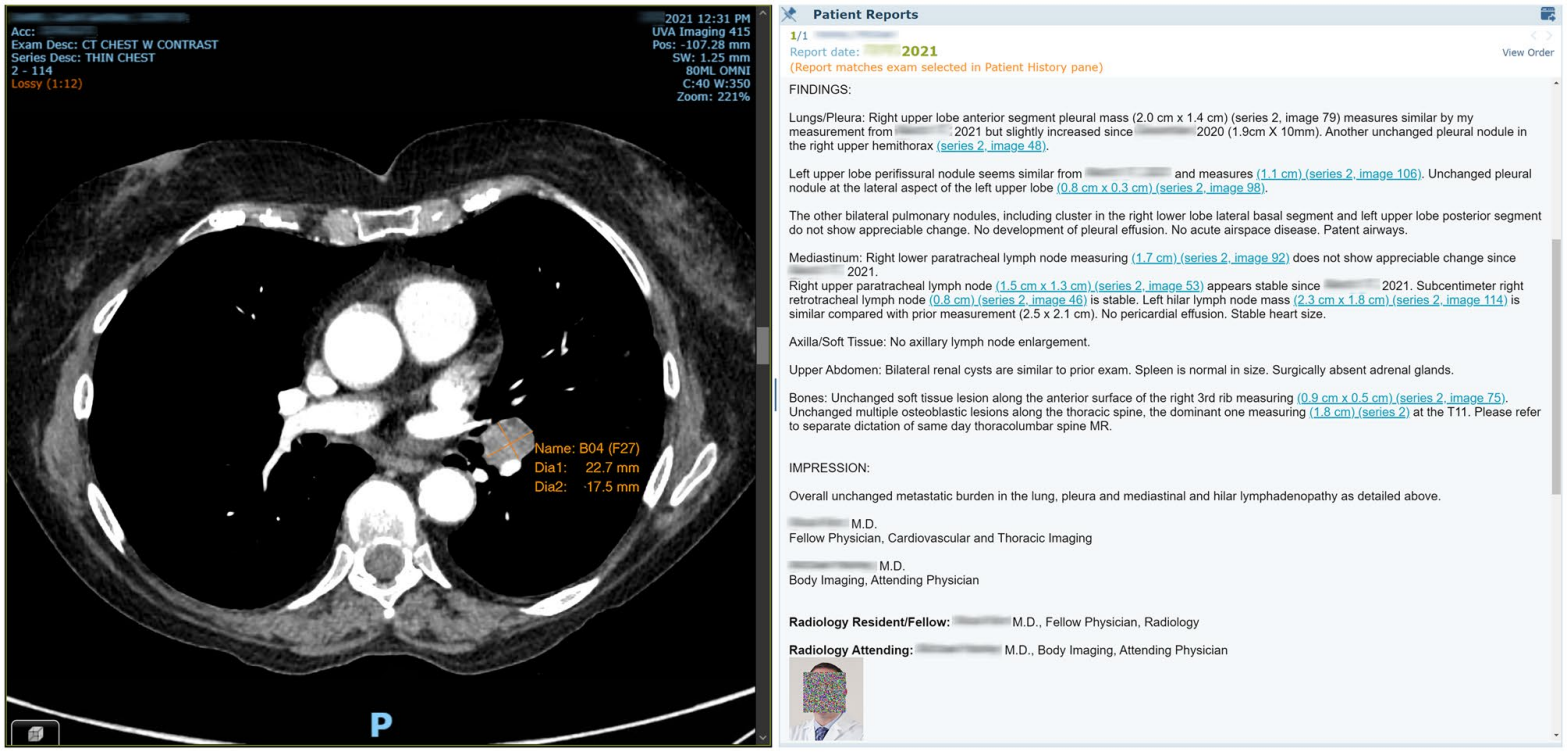
Example IMR in clinical use showing how a hyperlink in the report text will show the image of interest in the report viewer

In the same way that multimedia and hyperlinks have changed the way content is consumed on the Internet, multimedia reports for medical documentation have dramatically changed the way image-centric specialists communicate [4]. The elements of an IMR may be summarized into the categories of formatting, interactivity, and structure. Figure 1 shows a schematic representation of how an interactive multimedia report (IMR) encapsulates the relationship between text and images by context sharing during report authoring so that this context is unambiguous for the report consumer. Interactive multimedia reports have been shown to provide more robust communication with clinicians while reducing the ambiguity of findings [5–7]. Figure 2 shows an example from a commercial IMR implementation with hyperlinks from the report text linking description and visualization of a lung nodule. These innovative reporting solutions are transforming medical documentation from the age of the typewriter to that of computers or smartphones with efficient access to images, pictures, graphs, and diagrams.

In August 2019, the HIMSS-SIIM Enterprise Imaging (EI) community began the Interactive Multimedia Reporting Workgroup with a charter to advance reporting conducted by image-centric medical specialties [8]. By the fall of 2021, the workgroup consisted of over 80 medical specialists, informaticists, and consultants from cardiology, dermatology, endoscopy specialties, ophthalmology, pathology, physiatry, radiation medicine, radiology, and industry. The workgroup adopted a consensus definition of IMR as “interactive medical documentation that combines clinical images, videos, sound, imaging metadata, and/or image annotations with text, typographic emphases, tables, graphs, event timelines, anatomic maps, hyperlinks, and/or educational resources to optimize communication between medical professionals, and between medical professionals and their patients.” [9] Discussion in the workgroup included the current state, near future, and long-term goals of interactive multimedia reporting in all medical disciplines. Use cases from different medical specialties have been reviewed, including those implemented at multiple sites [4, 9–11]. An emerging theme has been the lack of interoperability between disparate EHRs, image archives, viewers, and report authoring tools which is a barrier preventing widespread implementation of interactive multimedia reporting.

Although the idea of interactive multimedia reporting is not new [12–17], the rapid growth of artificial intelligence (AI) in medical imaging has generated new enthusiasm for structured reporting and IMR. Interactive Multimedia Reporting, with clear digital links between descriptions and images, can serve as powerful ground truth for neural network training [18]. IMR may contain both narrative description and coded observations defined by ontologies. Creating semantic labels for imaging observations can be labor intensive for image-centric specialists. As AI systems play a greater role in image analysis, the imaging report will increasingly be a fusion between human and machine contributions [19]. Imaging reports must evolve beyond plain text summaries so that structured data can drive downstream workflows including critical result notification, follow-up tracking of specific image findings, radiology-pathology correlation, and patient-language summarization of reports.

**Fig. 3:**
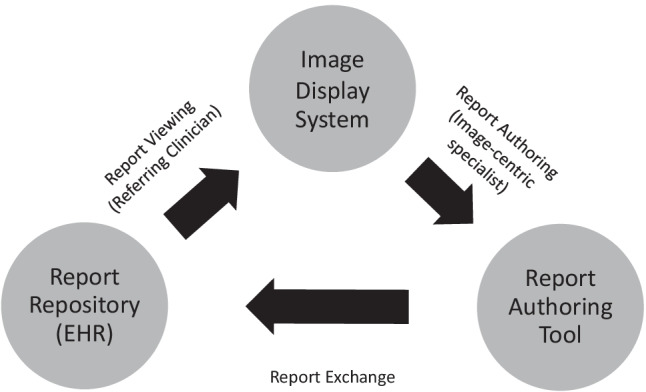
Graphical representation of the image-centric reporting cycle emphasizing how image-centric specialists use image display systems and report authoring tools to create reports that are then viewed by referring clinicians who then refer back to image display systems for correlation

This white paper focuses on the technical issues impeding multimedia report creation and integration with electronic health records. Examples are shown on how to overcome these challenges with technical development requirements including established and evolving standards, such as Digital Imaging and Communications in Medicine (DICOM) and Health Level 7 Fast Healthcare Interoperability Resources (HL7 FHIR). IMR may have many forms tailored to diverse use cases across different specialties. Standards must be adopted to enhance these workflow steps: (1) report authoring, (2) report exchange, and (3) report viewing (Fig. 3). The Integrating the Healthcare Enterprise (IHE) organization, which profiles technology standards to solve specific use cases, is currently drafting an integration profile to specify a standard approach for the exchange and viewing of IMR along with interactivity with image display systems [20]. This profile is necessary, but not sufficient, to bridge the current gaps that must be addressed so that IMR becomes a routine part of medical image analysis.

## Report Authoring

The report authoring process performed by an image-centric specialist varies greatly between medical disciplines. Radiology exemplifies the most mature and interoperable technical tools. The core elements of the workflow can be distilled into common themes across specialties. Diagnostic radiologists spend the bulk of their day at a picture archiving and communications system (PACS) workstation interpreting and reporting on imaging findings, but a surgeon dictating an operative note and citing intraoperative images captured during laparoscopic surgery may also function as an image-centric specialist in this scenario.

The specialist must first view the images to be interpreted. Images may be manipulated and/or processed into a new image. For example, the image-centric specialist or technologist may create annotations and make quantitative measurements. The image-centric specialist may use multiplanar reformation (MPR) or 3D reconstruction to create a new image demonstrating an abnormality to greater advantage. The image-centric specialist then describes the relevant observations in a report and ascribes meaning and diagnosis to the findings. Traditionally as part of this synthesis, the findings are compared across historical imaging studies and with consideration of other clinical data. A text report attempts to encapsulate the specialists’ thought process in words. The major advantage of IMR is the ability to more explicitly convey information with images, graphs, and tables. The report authoring process concludes with an impression summarizing the findings, drawing conclusions, and making recommendations. The finalized report is transmitted to the EHR, where other clinicians can view the report and determine the next steps in the patient’s treatment plan. Patients can also view their reports and images through EHR patient portals [21, 22].

In pathology, reports are almost universally authored in a laboratory information system (LIS). The LIS may exist as a standalone information system that interfaces to an EHR, or it may exist as a module within the EHR. In some instances, third-party add-on modules are interfaced with the LIS and contribute to the report creation. The most common type of add-in module is used to create structured data for synoptic reports, but other sources of report data input may come from middleware that provides molecular, flow cytometry, or image analysis results. In the near future, it is anticipated that AI modules may also contribute to pathology report authoring. Various forms of text and discrete data may be added to a pathology report at several points in the lifecycle of a specimen. Thus, the pathology reporting process must enable multiple contributing authors and data sources.

The report authoring process may look very different for image-centric specialists that are interpreting images in addition to their clinical evaluation. For example, a dermatologist may describe the findings of a skin lesion in the clinical note along with the history, physical, assessment, and plan. An encounter-based imaging workflow (EBIW) describes necessary steps to associate a collection of images, as perhaps captured by a digital camera in a dermatologists’ office, with the same encounter as the clinical note [23]. The description and images may be linked at the study level; however, common workflows may not support linked findings. Specialties such as dermatology, ophthalmology, and endoscopy require workflows and standards beyond those in practice and defined in EBIW.

### Report Content Contributors

#### Text Content

Text content in an imaging report may be created by typing, dictation, and transcription or imported from upstream information systems, image acquisition modalities, or post-processing tools. A report authoring system may receive an HL7 feed from a Radiology Information System (RIS) or LIS and import defined fields including patient demographics, exam indications, and study type. CT dose information is transmitted from the modality (e.g., CT scanner) as a DICOM Patient Radiation Dose Structured Report (SR) and may be parsed for inclusion in the report. Other DICOM SRs are often used to encode measurements made on an ultrasound (US) modality by a technologist prior to formal report interpretation by a radiologist, cardiologist, or obstetrician. The provenance of these measurements is lost when the data is sent to a text report; thus, the report reader does not know which source images were used to make the measurements. This workflow generally assumes a one-to-one mapping between a coded field from a modality and a structured field in the report template. There is no easy mechanism for resolving ambiguities or many-to-one relationships other than through time-consuming corrective processes. Most systems are incapable of handling unsolicited observations or uncertainties in measured findings (e.g., when variable numbers of nodules are measured separately). Some systems enable a user to adjust an existing measurement or decide which variation of a replicated measurement to use (e.g., maximum, minimum, last, average, or original). Systems have been designed to utilize a technologist’s input and further generate structured report content using established classification systems such as the TI-RADS criteria for thyroid nodules on ultrasound [24].

In anatomic pathology, a report may have multiple authors (e.g., residents, fellows, technicians, technologists, pathology assistants, and pathologists) populating sections of the report during specific steps in the workflow. Some complex specimens may require supplemental processing or testing. Additional authors and/or interfaced instruments may add results from biomarker analysis or molecular testing to the final report. Ultimately, while the report itself may look similar, the provenance of its content will vary significantly depending on the local implementation of laboratory workflows. In addition, although structured/synoptic reporting is widely adopted in pathology, text-based reports cannot sufficiently maintain the structure of useful data elements.

#### AI Results

AI systems are being designed to evaluate image features that may need to be included in the final imaging report. Current AI systems provide output in a variety of proprietary and non-standard formats. The IHE AI Results (AIR) profile seeks to standardize the mechanism by which AI systems communicate results [25]. Relying on the image-centric specialist to view the output of an AI tool and then dictate the results into a clinical report fails to achieve the workflow efficiencies that these new tools may provide. Nonetheless, it is essential that a mechanism be provided such that the report interpreter curates the AI findings before they are finalized in a signed report. With respect to AI results populating reports, the need for provenance is increasingly important. It is essential for a report consumer to know that observation was rendered by an algorithmic tool, which source data was used, and how the result was derived.

#### Source Images

One of the key elements of many IMR examples is a thumbnail image included in the report. Some multimedia report implementations use screen captures to include in the report. Most screen captures, however, have no link back to the source image and do not enable a report reader to click on a hyperlink to view the full-fidelity image in a dedicated image viewer, in context with the surrounding images. In order to generate an IMR with this interactivity, an imaging system must support unique identifiers (UID) of images, an application programming interface (API) to retrieve these images, and a mechanism to launch a full viewer to display these images in context.

The DICOM Standard (PS3.4) defines clear guidelines for retrieval of images and annotations from a PACS or vendor neutral archive (VNA) that are commonly used in radiology, cardiology and obstetric departments [26]. Other disciplines such as dentistry, pathology, dermatology, ophthalmology, and endoscopy often use proprietary systems to capture, store, and display images that may not be standardized. The debate of whether all medical images should be internally stored in a DICOM-compliant archive is beyond the scope of the present paper. Certain prerequisites must be met in order to have a meaningful discussion on how images can be integrated into reports in a vendor neutral and interoperable mechanism. For example, an imaging system may store rendered JPEG images on a file system indexed by a proprietary file naming convention. A reporting tool has no vendor neutral means to refer to these images within an IMR without a standard interface. Worse yet, some isolated image capture devices, such as in ophthalmology, may not have any network connection to transfer images to other systems. The American Academy of Ophthalmology recently released a position statement advocating vendors to adopt the DICOM standard [27]. Grassroots efforts like this are needed to provide the foundation for IMR across image-centric specialties.

**Fig. 4:**
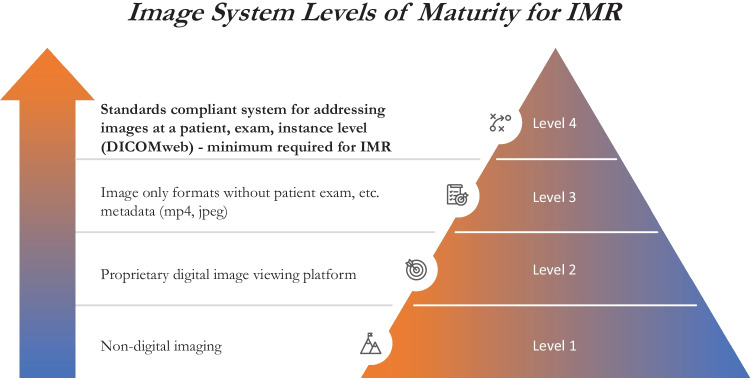
Broad categories of imaging systems presented in a hierarchy of sophistication toward supporting vendor neutral interaction with IMR (level 4)

The DICOM Standard defines a system of UIDs (PS3.5) and an information model that unambiguously refers to studies, series, and instances within an imaging study (PS3.3) [28]. A group of related images may be stored as a series of instances, as is common in CT and MRI, or as frames within a multi-frame instance, as is more common practice in ultrasound. The standard also specifies DICOMweb (PS3.18), a modern mechanism for the retrieval of images [29]. A uniform resource locator (URL) is constructed using the UIDs of the study, series, instance, and/or frames of interest. The specific image(s) can be retrieved either as a DICOM object or rendered as an image or movie in formats including JPG, PNG, GIF, MPEG, MP4, or H265. If the specific UIDs are unknown, DICOMweb also defines query functionality. The FHIR ImagingStudy resource defines a compact representation of key metadata for all the instances within an imaging study [30]. Importantly, the FHIR resource refers to series and instances by their DICOM UID and relies on DICOMweb for actual retrieval of images. The proposed levels of maturity, inspired by the Digital Imaging Adoption Model [31], describe how a given system may store images, with the ultimate goal of supporting IMR (Fig. 4). DICOMweb support is the minimum requirement of an imaging system to support vendor neutral IMR creation. It is imperative that vendors not only support DICOMweb interfaces but also provide APIs to integrate systems without onerous costs.

In pathology, issues with digital image compatibility and retrieval remain a significant barrier to interoperability. Digital images of various types can be captured at various steps in the pathology workflow, and these typically reside in vendor-specific platforms that often lack standardized mechanisms for image transmission. Whole slide imaging (WSI), the process of creating a single, large high-resolution digital image from a glass slide that contains microscopic images of histological or cytological nature, is the most contemporary and well-known form of imaging in pathology, but it is not yet widely adopted in clinical practice. Support for the DICOM standardized representation (PS3.3) for WSI continues to grow among digital pathology vendors [32]. While WSI gets more press, the most common type of digital images in pathology are photographs taken during specimen dissection using digital cameras. These “gross photographs” supplement the textual description of specimens and serve to document the size, location, and appearance of macroscopic findings such as tumors and other lesions. Although interoperability could be achieved with DICOM, it is rarely used for gross photography. The same is true for other types of clinical imaging in pathology, including brightfield photomicrographs, fluorescence photomicrographs, and transmission electron micrographs. At best, these digital images will be collected into a laboratory information system (LIS) or stored in a standalone image repository, but rarely do they end up in a PACS, VNA, or other DICOM-capable repository.

The common approach of indexing to the DICOM instance level is sufficient for many scenarios but may be inadequate for others. For example, the image of interest may have a specific zoom level and field of view within a larger WSI data set. Image indexing is also challenging in modalities that encode other types of media. Video that may be captured during surgery or endoscopy is often compressed in a lossy manner so that individual frames cannot be referenced. Commonly, a reference to a specific time point in the file is needed when referring to video or audio content. DICOM Structured Reports (defined in DICOM PS3.3) allow for a temporal coordinate (TCOORD) value type that might be better suited for a video reference. As dynamic 3D manipulation of datasets becomes more common within viewers for CT and other high-resolution cross-sectional imaging, the key image may be dynamically created. A dynamically created image may be saved as a secondary capture instance. However, this mechanism is like screenshot creation and loses much of the value of the image reference in an IMR. Instead, a custom multiplanar reformation of a 3D data set could be saved as a DICOM Planar MPR Volumetric Presentation State (DICOM PS3.3).

#### Annotations

In the course of interpreting images, an image-centric specialist will view source images and create annotations to indicate the location of findings and quantify disease. The specialist may make a quantitative measurement of the image such as linear distance measurement, circular region of interest (ROI) with average pixel value, or a 3D volume quantification. The specialist may add annotations such as an arrow, oval, or text box to highlight and explain findings within the image itself. The specialist can flag specific images as key images of importance. Although the basic annotation tools are similar across disciplines and software packages, implementation details vary widely.

Basic annotation forms may be non-digital such as a ruler or wax pencil marking placed on a pathology slide that is captured in a digital photograph. Some digital tools “burn-in” annotations in the source images (e.g., ultrasound systems), thus preventing the viewing of images without the annotation. Other tools may implement their own proprietary schema for encoding annotations that are incompatible with other systems. The DICOM Standard describes several mechanisms for defining annotations in vendor neutral format [33]. Clinical systems most commonly use DICOM Grayscale Softcopy Presentation State (GSPS), or its color equivalent, to encode and transmit many types of graphical annotations.

A presentation state describes how to encode various display parameters of an image, including the graphical content of the annotation. Presentation states do not encode any semantic explanation of the finding. A DICOM Structured Report (SR) encodes 2D, 3D, or temporal coordinates, measurements, and findings, together with coded labels. A key object selection (KOS) object is a subtype of SR that references specific DICOM instances “of interest” along with textual or coded labels. The creation of rich semantic markup (graphic coordinates or segmentation of an annotation together with the explanation of its meaning) is often labor intensive, requiring users to use structured data capture drop boxes instead of voice recognition systems [34]. Beyond the simple graphic primitives that are used as clinical annotation, the DICOM Standard (PS3.3) defines more sophisticated forms of annotation that may be encoded including formats to describe image feature segmentation (SEG, DSO), radiotherapy structure sets (RTSS), real-world value maps, and parametric maps. These rich pixel annotations are very time consuming to create manually and are, therefore, not often created in the clinical workflow. However, the advent of AI segmentation tools may make these types of annotations more feasible in clinical use for inclusion in an IMR.

The linking of image findings coded with a semantic annotation may facilitate the correlation of radiology and pathology findings as a longitudinal history of the disease. Currently, tools that enable this require semi-automated intervention to link specific observations over time. Patient-level semantic annotation has the potential to link pathology reports to radiological images documenting locations of biopsies and/or surgical specimens. The new Radiology Pathology Concordance IHE profile describes how discrete data elements are collected from structured reports to create an integrated report, where concordance of results is assessed, and reports are shared to an EHR or information system in use by a health facility [35]. The integrated report would extract data from structured radiology and pathology reports, compare the extracted results, and assign a score based on the concordance of the results which will be included in a new integrated report.

Tools exist to construct patient-specific timelines of images and other diagnostic test results [36]. Due to the lack of standardized data, images must be tagged with metadata referenced to an ontology using natural language processing (NLP) or manual labeling. In the absence of semantic annotations that link image observations to a finding, images must be manually dragged and dropped to connect images with a specific finding. The result is an IMR with related multimedia content linked in timelines that illustrate the natural history of the disease.

**Fig. 5:**
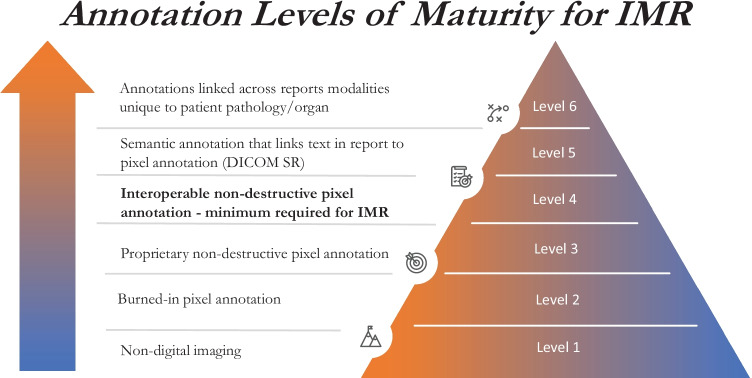
Broad categories of annotation systems presented in a hierarchy of sophistication for supporting vendor neutral IMR (level 4) and additional use cases of semantic annotations such as radiology-pathology correlation (level 6)

Proposed levels of maturity categorize how a given system may store image annotations and are presented in Fig. 5. In order to link the context of an image annotation with a report, the annotation must conform to one of the standards, such as the DICOM Presentation States or Structured Reports, that describe annotations that are not burned into the image (non-destructive annotation). The workflow of dictating measurements manually in a report is time-intensive and prone to human and/or transcription errors [37]. Annotation standards facilitate the transfer of measurements into a report [38]. These standards can also encode the semantic meaning of these image annotations. The report authoring workflow must evolve, perhaps augmented by AI tools, to enable the capture and documentation of more meaningful semantic annotations.

### Report Authoring Process

The key technical barrier in the reporting workflow is the absence of a standard, vendor neutral approach to instantaneously communicate image observations to a report authoring tool. As an example, a radiologist may identify and measure a lung nodule in a chest CT on a PACS viewer while simultaneously dictating the measurement, image location, and appearance in a voice recognition system. To create an IMR, the unique identifier of the image must be communicated to the report authoring system so that a link to the image can be incorporated into the final IMR. Real-time communication of measurements from the PACS can reduce the burden and potential error when creating reports by only dictation. Creating a measurement in a PACS display can be transmitted in real-time to the reporting system for inclusion in the report as both text and a structured field [5, 38].

Improvements to this process have been offered by the vendors of reporting systems through the introduction of proprietary APIs, allowing the automated transfer of information to be included in the text of the report. A PACS could leverage these APIs as means of integrating with the reporting systems. Commonly, the insertion of the data is done at the current position in the text of the report when the user explicitly selects to insert specific data elements by pressing a button or issuing a voice command in the user interface. The current implementations are an improvement over manual typing or dictation but have a number of drawbacks:

- Proprietary nature of the APIs limits the interoperability and types of data that may be exchanged and requires larger effort for development and maintenance.
- Measurement values that are captured by a PACS are usually lacking the semantic context in which they are defined – for example, two perpendicular measurements of the long and short diameter of a lesion are transferred as coordinates, and their exact meaning is defined by the textual narrative in the report. Thus, even with the automatic transfer of data, semantic errors may occur when measurements are inserted at the wrong position in the report.
- Although the PACS can provide hyperlinks to images as data being submitted via such APIs, the use of such hyperlinks is limited, as the reporting systems or downstream report viewing systems do not usually provide the ability to navigate through those hyperlinks and access the imaging content.

Alleviating these drawbacks will require a higher level of interoperability so that all participating systems can reliably generate, exchange, interpret, and render the complete multimedia content of the report. A new type of interface is needed to achieve this functionality outside of a proprietary single-vendor image viewing and reporting system.

#### Real-time Communication Between PACS and Reporting System

As discussed above, a key element for an IMR is the ability to include clinical findings such as measurements and ROI with interactive links to the source images. Traditionally, these annotations, markups, presentation states, and key images could be captured as DICOM objects such as GSPS, SR, or KOS. These objects are designed to capture evidence for long-term reference instead of real-time communication or composition. Most PACS will create these evidence objects at the end of a session in order to capture all the data points created by the image-centric specialist in one object, rather than create multiple evidence objects resulting in one per data point. As a result, these evidence objects in DICOM are good resources for subsequent interactive access when viewing an IMR, but not good candidates as the payload for real-time communication during a reporting session. As the image-centric specialist captures measurements, regions of interest, and other data points, the PACS should provide those data points to the reporting system in real-time without introducing any unnecessary interruptions or adding transitory content to the permanent record.

**Fig. 6:**
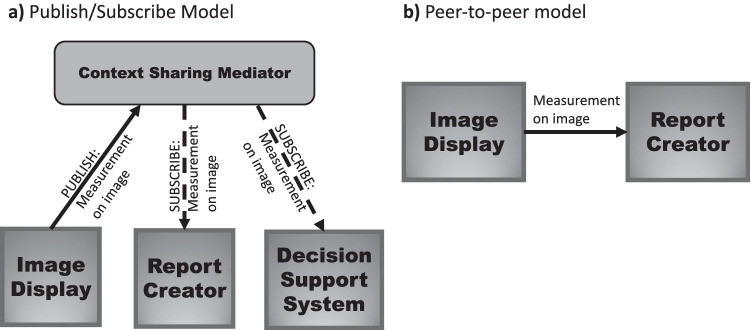
Models for real-time communications channel between an image display system and report creator. In the Publish/Subscribe model (**a**), a system can publish a message to the mediator (solid line). A message might be triggered by an event in the image viewer, such as creating a measurement and marking a key image. Additional systems that have subscribed to receive this type of message will receive the payload (dotted lines). In the peer-to-peer model (**b**), a transmitting system communicates directly with the receiving system

The design for these real-time communication channels usually comes in two versions; the publish/subscribe and peer-to-peer models are depicted in Fig. 6. In the publish/subscribe model, a topic is created for the publisher system to publish messages. Consumer systems who are interested in the topic create their own subscriptions to the topic in order to receive notifications. A mediator sits between the publishers and subscribers and is responsible for managing the topics and subscriptions to ensure messages on a given topic are delivered to all the subscribers. In a peer-to-peer model, there is no mediator; the publisher is directly connected to the consumer. There are pros and cons to these two models. The publish/subscribe model scales well when there are many publishers to many subscribers. However, it requires a more complex infrastructure and is consequently more complex to set up. A peer-to-peer model is more straightforward to get started, but it is less scalable.

Several technologies could be used to implement an efficient communication channel between an image viewing system and a report authoring system. For example, HL7’s FHIRcast uses the publish/subscribe model [39]. It uses the WebSub W3C standard [40] and originated as an application context synchronization standard that extends the SMART on FHIR authentication pattern. When applying FHIRcast in the context of IMR, the PACS can publish messages regarding the exam (e.g., identifier of a radiology exam) as well as granular context sharing of specific images and measurements (e.g., measurement details and referenced images), and one or more systems can subscribe to this notification and respond accordingly; in particular, a dictation system might launch in an exam context, as well as insert the measurements or image references during dictation. The message payload, or event, in FHIRcast is a FHIR resource, such as an observation.

For scenarios when both the image viewing system and report authoring system are webapps, FHIRcast may be too heavyweight due to the need for a mediator. The HL7 Context Management Specification predated FHIRcast and was never widely adopted, partially due to the need for a “context broker.” [41] The proposed SMART Web Messaging standard is a lightweight alternative that can transmit the same event payloads as FHIRcast, over HTML5 (HyperText Markup Language) web messaging [42]. This enables two systems to communicate in the same browser process using the same payloads that FHIRcast integration uses, thereby enabling the developers to integrate rapidly, and then progressively enhance the solution toward FHIRcast without modifying the event payloads.

It is important to note that this real-time communication is not limited to IMR but generally applicable to many contexts. A real-time mechanism to share granular context from an image viewing system could create a plug-in architecture for decision support systems to extend the image analysis and reporting process. In our prior example, an AI system might be notified of a new measurement, pull the measurement source image, and automatically identify the anatomic location and Lung-Rads score that could then be transmitted to the reporting system.

#### Security Concerns in Report Authoring

As with any communication between two or more clinical systems, security is a critical part of the design consideration. There are three areas of particular concern to reporting to consider: (1) identity and authorization, (2) communication confidentiality, and (3) data integrity and availability. In terms of identity, one may implement a zero-trust model to validate whether a publisher or subscriber is authorized to transmit or receive the data. When establishing communication between systems, the choice of encryption methods and protocols should be sufficient to ensure the confidentiality of the data is protected while in transit to prevent a compromise should the communication be intercepted by malicious threat actors. Both the transmitting and receiving systems must be able to validate the integrity of the data during transmission. Furthermore, to avoid disruptions to services, best practices such as (but not limited to) web application firewalls (WAF), load balancers, and data loss prevention (DLP) systems should be utilized as appropriate between the systems communicating.

## Report Exchange

For an IMR to be of clinical value, the report must be transmitted to downstream systems (e.g., EHR) where clinical consumers can view it. The report should also be transmissible and viewable, ideally with preserved functionality, across institutions that may not share the same EHR. An IMR can take several forms depending on the use case and complexity and exist on a continuum of structured reporting [43]. IMRs can exist as free-form narrative text, structured/synoptic reports, or some combination. Although free-form narratives can be organized with templates containing headings and subheadings, a truly structured report is a report in which coded observations are associated with a coded field according to an ontology such as SNOMED CT or RadLex.

Synoptic reporting is another term for structured reporting in which all findings are described as discrete data elements [44]. A move toward synoptic reporting emerged in pathology. Common data elements (CDE) have been defined and are required when reporting on specific study types [45]. These CDEs can be sent in a coded fashion, so they are easily readable by a computer system. Both HL7 V2 and FHIR standards offer the ability to encode structured values together with the text report. IMRs containing structured observations (e.g., CDEs) can drive downstream workflows and be used for training and continual improvement of AI algorithms.

Despite a move toward increasing structure during the reporting process across various image-centric specialties, the narrative report remains essential to convey the nuance, ambiguity, and clinical gestalt inherent in the practice of medicine. An IMR often contains both structured and narrative content which can be combined in several ways. The top-level messaging format may have coded content and a specific section for the narrative report. In addition, the narrative report itself may be in a tagged format, such as HTML or XML (Extensible Markup Language), that permits the embedding of structured content at the word or sentence level. The hallmark of an IMR is that the narrative text content is mixed with images, diagrams, and hyperlinks within. Different types of messaging and report presentation formats are discussed that can bundle structured content with a report payload.

### Messaging Formats

In order to exchange a report between software systems (e.g., report creator and EHR), the report must be bundled with descriptive metadata and transmitted in a specific format using agreed transport protocols. The messaging formats described here specify both the format of the message as well as the transport protocol to be used. Discussion of transport protocols is out of scope for this paper, but standardized transport is essential for report exchange.

#### HL7 V2

At present, most clinical reports are transmitted in HL7 V2 ORU messages from a reporting system to receiving systems such as the EHR and PACS [46]. HL7 V2 is an interchange format that combines structured metadata about the report and the report itself. The HL7 message is composed of different named segments on separate lines; each segment is pipe-delimited into numbered fields. The clinical report is contained within an observation segment (OBX), and the report content may be transmitted as plain text in the message or as a Base64 encoded attachment. In addition to the text of the report itself, HL7 messages may also contain additional OBX segments that define coded observations. The North American Association of Central Cancer Registries (NAACCR) uses this approach to define a standard for transmitting cancer synoptic reports as structured data [47]. Despite the ubiquity of the HL7 V2 format, many downstream systems, such as EHRs, are unable to interpret such structured reports; a communication standard is only as good as the conformance of receiving and transmitting systems. In practice, laboratory information systems send the structured report to cancer registries and data warehouses and send a stripped-down HL7 V2 message with only the narrative report to the EHR.

#### HL7 FHIR

HL7 FHIR are a set of modular resources which may be bundled and linked to each other to communicate complex information in a healthcare setting. The FHIR DiagnosticReport is a resource that may serve as a container for a report and additional resources that may include attached images or discrete data elements coded as FHIR Observations. A DiagnosticReport may be combined within a Bundle with a Composition, which defines how the various discrete data elements fit together. Like HL7 V2, the FHIR DiagnosticReport may include a presentedForm that is a Base64 encoded representation of the narrative report in one of the report formats below. Alternatively, the narrative report may be described within the FHIR Composition.

#### DICOM SR

The DICOM Structured Report (SR) family of objects (PS3.3) has been widely adopted in clinical practice for encoding data during the exam acquisition process such as dose data from a CT exam or measurements from an ultrasound. DICOM SR specifically separates semantics from the presentation and does not support the inclusion of preformatted text. However, an SR report can reference other DICOM objects including images and DICOM-wrapped PDFs. SR files are transported using DICOM transport mechanisms that are widely supported by PACS but less so by EHRs. Despite the success of DICOM SR as a format between modalities and PACS, the adoption of DICOM SR as a messaging format for transmission between report authoring systems and EHRs is thought to be unlikely.

### Report Presentation Formats

The messaging formats above may be sufficient to encode a purely synoptic report which contains a series of coded observations. However, an IMR is characterized by a narrative that mixes richly formatted text with tables, images, diagrams, and links to describe the exam findings. These formats are wrapped in the containers above and must be parsed and displayed in the receiving EHR, PACS, LIS, and/or RIS. Each format is rated along with various categories in Table 1.

**Table 1:** Report presentation formats

**Report format**	**Formatting**	**Images/video**	**Responsive**	**Fixed rendering**	**Interactivity**	**Programmability**	**Structure**
Plain Text	-	-	+/- ^a^	-	-	+ +	-
PDF	+ +	+ +	-	+ +	+	+	-
HTML	+ +	+ +	+ +	-	+ + ^b^	+ +	+ ^c^
RTF	+ +	+	-	+ +	+	+	-
Wikitext/Markdown	+	+	+	-	+	+ +	-
FHIR Composition^d^	+ +	+ +	+ +	-	+	+ +	+ +
CDA^e^	+	+	+	-	+	+	+ +

Relative comparison of different formats that may be used to capture an interactive multimedia report. Note that formats must support basic formatting in order to be included. Purely semantic representations are excluded. Plain text is included as a reference. Each category is rated on a 3-point scale

- not supported, + supported with basic functionality, + + supported with advanced functionality

Categories:

• **Formatting:** allows for text decoration, fonts, page layout, tables, and other visually rich displays of text;

• **Images/video:** incorporates these forms of multimedia into the report;

• **Responsiveness:** adapts to different screen sizes and form factors;

• **Fixed rendering:** the inverse of responsiveness and characterizes how well the format maintains its rendered appearance across devices;

• **Interactivity:** ability to support user interaction. Level 1 describes formats that can encode basic hyperlinks; level 2 formats offer even richer interaction (e.g., through widgets containing buttons, sliders, and other UI elements);

• **Programmability:** relative measure of how difficult it is to create or alter the format using common technology stacks

• **Structure:** incorporates coded observations. Note that the messaging format supports coded observations at the report level, but the reporting format may also support coded observations at the individual word or phrase level

^a^Plain text with embedded line breaks is not responsive.

^b^Most formats provide basic interactivity via hyperlinks. HTML supports richer interactivity via JavaScript.

^c^It is technically possible to mix semantic labels in HTML tags surrounding text, but there is no standard to do so.

^d^FHIR composition uses XHTML for display, encompassing many of the benefits of HTML with more limited interactivity and greater structure

^e^Requires transformation into another format for display such as RTF, PDF, or HTML

#### Plain Text

A plain text report is a string of ASCII or Unicode characters as one might create using a typewriter. Only minimal formatting is provided in a text report by new line and tab characters, and emphasis is often conveyed by changes in capitalization. Various factors at the discretion of the display system determine whether content alignment and line breaks are the same as on the authoring system. These factors include font, character spacing, width of display window, and tab character width. The chief benefit of plain text reports is that they are generally passed between systems without degradation since they are almost universally supported.

#### PDF

Portable Document Format (PDF) is a proprietary format developed by Adobe during the early 1990s, based on the PostScript format. Adobe made PDF available for free use in 1993, and in 2008, control of the format was assumed by the International Organization for Standardization (ISO). PDF is designed to display documents to appear the same regardless of the device (e.g., printer, computer display, mobile phone), operating system, or application used to generate and display the document within the limits of the device’s capability. PDF is commonly viewable through commercial EHRs due to integration with dedicated document viewers. PDF can also be encapsulated in DICOM (PS3.3) for distribution in the PACS or archival in a VNA.

#### HTML

HTML is the widely known text-based format used to encode web pages. It supports complex layout, hyperlinks, multimedia, varying fonts, and document styling. HTML may be coupled with JavaScript, a programming language that powers much of the interactivity experienced in a web browser, to support complex interactive applications. HTML reports can be viewed in web browsers or in other applications that support HTML display, including many EHRs, RIS, and imaging viewers. In many uses, HTML documents depend on content beyond the single HTML document file such as stylesheets, media, or image resources. Since the report document is not self-contained and referenced resources may exist on systems with different lifecycle management policies, the resources may be modified or may become inaccessible after time, known as “link rot.” The use of HTML for IMR may work best if a prescriptive approach to embedding resources is taken, for example, with data URLs for images and self-contained style elements.

#### RTF

Rich Text Format (RTF) is a proprietary document format developed by Microsoft in the late 1980s. It supports hyperlinks, multimedia, multiple fonts, and text styles. Multimedia is self-contained within the RTF file. Support for RTF features differs between Microsoft products and other third-party products. RTF has support in many EHR systems and is relatively easy to generate programmatically because of its text-based approach. However, because RTF has never been truly standardized, it can be very difficult to ascertain which features (e.g., tables) are supported by each EHR.

#### HL7 CDA

The HL7 Clinical Document Architecture (CDA) is a text-based standard for specifying structure and semantics in XML [48]. XML is a text-based format like HTML. While HTML primarily encodes presentation, XML is used to encode arbitrary structure or meaning. As an XML-based format, CDA does not specify visual formatting and must be transformed using Extensible Stylesheet Language into a rendered report. CDA can support a wide variety of document types and can be exchanged in a variety of ways, including over HL7 V2 and as a standalone document. The CDA body contains the clinical content of the document in either a structured or unstructured form. The body can optionally contain Base64 encoded content, including RTF and PDF. A CDA can serve as both a messaging format and a markup format. A template for diagnostic imaging report content (CDA implementation guide) and transform between DICOM SR and HL7 CDA documents is specified in the DICOM Standard (PS.3.20). There has not been large-scale adoption of this format for imaging reports to date.

#### HL7 FHIR Composition

The Composition resource describes a report composed of multiple sections. Each section can contain a text element with a description of the section text marked up with limited XHTML including links, images, and internally contained style attributes. XHTML is a restricted version of HTML which, among other differences, prohibits JavaScript active content, improving security at the expense of interactivity.

#### Other Formats

In addition to the above formats, IMRs might be encoded in modern markup languages that have been widely adopted in other domains. Examples include Wikitext, which is used in MediaWiki projects (such as Wikipedia) and Markdown, which has been implemented widely in web-based applications, most notably at GitHub. Both Wikitext and Markdown were designed to be written and read directly by humans but are usually rendered into HTML for display purposes. A key design principle of Markdown is that the document should be easily viewable as plain text without the syntax interfering with its readability. FHIR uses Markdown for certain elements where some level of formatting is desired, but full XHTML support is deemed excessive.

### Security Concerns in Report Exchange

Report exchange requires consideration of many security implications. The details of securing a communication channel between a report authoring tool and EHR are out of scope for the present discussion. As each system or data source is connected, data integrity, authentication and authorization, infrastructure, and presentation device security should be appropriate for the lifecycle of the data with minimum standards defined before a system can be connected. A legacy system utilizing a deprecated encryption method would introduce a potential risk to other connected systems such as the report authoring tools and/or the EHR. Ensuring encryption used is based on appropriate ciphers and strengths for the messaging and transport protocol, such as standardizing on TLS1.2 or TLS1.3 for HTTPS, would increase the overall security of the information exchange.

Some of the formats discussed create new threat surfaces that do not exist in plain text reports. PDF files can contain scripts to facilitate extended capabilities but could be hijacked to embed a virus. JavaScript embedded in HTML can enable interactive widgets as it does on the Internet. Running such code requires a degree of trust in the development, maintenance, and security of the code and the device used to view the report. The exchange of such rich reports may require the cryptographic signing of reports or other mechanisms to ensure the integrity of the report from origin to the recipient, but such approaches have been hampered by the lack of widespread deployment of a certificate distribution and signature verification architecture. The authentication and authorization of both the creator and recipient of the report by multiple systems will be critical to maintaining the confidentiality of the data across many different user roles and access methods. Report formats that retrieve either the main report or secondary content from uncontrolled remote locations may be susceptible to retrieving malicious content or to link rot.

## Report Viewing

When viewing an imaging report, healthcare providers, and increasingly patients, are trying to understand the summary of findings to determine a disease state and actionable next steps in patient care. Optimal planning of the next step may involve referencing the images guided by the interpretation of the image-centric specialist. Direct viewing of the images may help plan a surgical approach, monitor and evaluate therapies, educate a patient, or allow a referring healthcare provider to form their own opinion of the imaging findings.

### Image Context Links

An interactive multimedia report may include embedded key images and other graphics such as graphs or diagrams presented directly with the text. However, the hallmark of the interactive multimedia report is interactive functionality, usually based on HTML hyperlinks embedded in a report that can launch a dedicated image viewer to inspect the designated location in the source images directly. For example, a CT image would be presented in the context of a set of scrollable images, or a zoomed pathology field of view would be presented in the context of the whole slide image. This direct linkage removes all ambiguity about the finding referenced in the report and allows the clinician reading the report to further interrogate the source images, guided by the interpretation of the image-centric specialist.

Reports may launch from different points and interact with different image display systems. For many users, reports are predominantly displayed within an EHR and will link to an enterprise image viewer. The EHR must be able to render the content of the IMR so that the formatting matches what was intended by the authoring process. Image-centric specialists may also view a report within their image viewing environment, such as the PACS, LIS, or WSI system, and expect that the hyperlinks launch images in their diagnostic viewer to facilitate a comparison of a current study to prior exams. An IMR may also be viewed in a dedicated report viewer (e.g., one launched from the EHR) that may add additional interactivity beyond that available in the EHR. In the USA, reports are required by law to be available to patients electronically [49]. Similarly, an IMR may be available to patients via a portal, along with images and hyperlinks [22]. Lastly, the IMR may be exported beyond the boundaries of the Health IT ecosystem, perhaps after encapsulating into a single file (e.g., a PDF report emailed to an out-of-network provider). To view IMRs, consideration must be given to support of interoperability requirements for several use cases including departmental access, intra-enterprise access through an EHR, and inter-enterprise access for users outside the creating institution.

Broad adoption of IMR will require industry consensus on file formats that can properly encode the documents. A standard is also needed for hyperlinks that can launch the source imaging system in context. The IHE Invoke Image Display (IID) [50] profile provides a common URL scheme for invoking a viewer in an exam context, such as when the link is clicked in an EHR. The profile specifies requirements for the display to provide interactive viewing of a complete set of diagnostic images, if requested. The interactivity required includes windowing, zooming, and panning as well as image navigation. Security is addressed through normal HTTP mechanisms. The display can take the form of a browser-based viewer, a separate applet, a plug-in or thick client-based viewer, or even a separate physical machine; the invocation remains the same and is agnostic to the viewer implementation mechanism.

The IMR IHE profile will extend IID to define viewer launch to display a specific image instance, presented in the context of its parent series. Image viewing systems must conform to a standard API for opening an image instance in the context of its parent when appropriately launched. For example, a thumbnail image in a multimedia report may represent an axial image which is a link to a full viewer. When clicked, the user should be presented with the full resolution axial image represented by the thumbnail image within a scrollable viewer that allows the user to see adjacent anatomy present in the DICOM Series. This functionality requires a new (or extended) standard and broad adoption across vendors.

### Special Image Viewing Situations

In the scenario of departmental access, an IMR may be viewed by a specialist in a dedicated system. For example, a radiologist may open an IMR within a PACS viewer, a pathologist may open an IMR within an LIS, and a clinician may open it within the EHR. Thumbnail images in the IMR may be hyperlinks to specific pathologies. When an IMR is opened in a radiology PACS, the radiologist would expect the hyperlink to navigate to a PACS viewport indicated by the thumbnail image of interest and in the context of the parent image set, thus allowing them to scroll through the surrounding images with cross-linking, and references across multiple image series. A pathologist viewing an IMR might click a hyperlink that opens a WSI viewer that displays the specific field of view depicted in the thumbnail image. Such deep integration is possible if the enclosing application intercepts the URL. Rather than launching the URL in a web browser, the path of the URL is parsed, and the content is interpreted by the viewing software to drive the image display. This workflow is analogous to the process of deep links in a smartphone operating system. Smart phones can intercept URL links to launch native applications to provide a richer experience for users who have access to a particular native app, while maintaining compatibility to launch a web application for users that do not have access to the native application.

The expectations for an IMR shared outside of the originating institution are less clear. It would be challenging from a security perspective for a hyperlink to direct the user back to the originating institution’s enterprise image viewer. Many organizations do not have an image viewer accessible to authorized users outside of the institution’s firewall. The receiving caregiver may not be authorized to access the enterprise viewer. An access code could be embedded within the report, but such an approach raises security risks. Similarly, what is the expected behavior when an IMR is ingested by another institution’s EHR? Should hyperlinks point back to the originating viewer, or should the hosting EHR intercept the URL and attempt to display the images in the local enterprise viewer? What is the expected behavior if the images do not exist in the hosting institution’s PACS or VNA? Should URLs within an IMR be altered when an IMR is transferred between institutions? A possible solution to these challenges is for an IMR to gracefully reduce functionality when shared between organizations. Thumbnail images could be bundled with the report, as in an FHIR DiagnosticReport, before the report is shared. Alternatively, a report could be rendered in a fixed format such as PDF, encapsulating the multimedia prior to transfer though it negates many of the benefits of IMR.

### Security Concerns in Report Viewing

The interactivity of an IMR raises several security concerns for viewing not present with text reports. Hyperlinks in an IMR provide context to launch an image viewing application. The image viewing application must verify that the user is authenticated and authorized to view those images. These functions could be provided by the EHR or other systems displaying the report. The interactivity also raises the possibility that links could be replaced maliciously. Just as a phishing email might direct a user to a malicious website to harvest personal information, a malicious IMR might redirect a user to an image display server hosting malicious code. These threats may be mitigated by a zero-trust model. In a zero-trust model, authentication and authorization are reassessed on each instance of displaying the report based on the context of the user, the device they are accessing from, certificates, form of authentication used, and potentially other variables related to the access of the report. For example, a user may have access to dermatology images for a patient but not surgical images of a specific surgical site such as gender reassignment. Using a zero-trust model would enable greater control over the specific data presented in a report.

## Discussion

Image-centric specialists communicate diagnostic and interventional imaging findings to clinical colleagues and patients. Imaging findings may automatically drive downstream workflows or business logic in a healthcare organization. Interactive multimedia reports facilitate the communication between an image-centric specialist and report consumers. The IMR intends to advance the traditional descriptive text report which may contain bias inherent in the image-centric specialist’s interpretation. The IMR will enrich the diagnostic report with ease of access to source data and related documentation. Ultimately, source images contain rich information that may further inform clinical judgment. Integrating multimedia into a diagnostic report can facilitate clearer communication of the disease process to consumers (i.e., clinicians and patients) and provide enhanced clinical care. An IMR reduces the guesswork when interpreting an image report by providing the narrative description integrated with tables, images, diagrams, and hyperlinks to the source images to better illustrate what is being described by the image-centric specialist. Automated transfer of image measurements and locations into an IMR combined with visual confirmation of what is being reported via multimedia has the potential to significantly improve patient safety, in addition to facilitating report creation. IMR streamlines communication between providers during tumor boards. When multiple providers collaborate on a diagnostic interpretation, the IMR facilitates collaboration (e.g., during digital sign out in a pathology environment). Education, clinical correlation, radiology-pathology correlation, quality assurance (QA), and patient-physician communication are all facilitated by IMR. Structured content within an IMR supports training and continual improvement of AI algorithms. IMR enables reports to transcend specialty-specific applications and bridge the gaps between image-centric specialties and their report consumers.

**Fig. 7:**
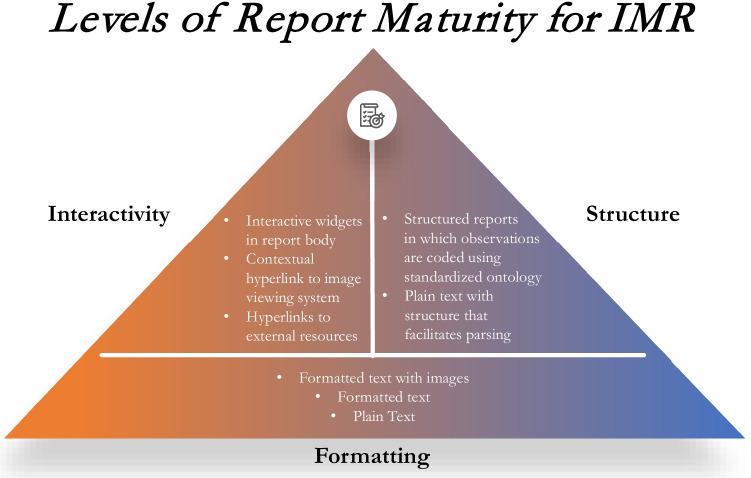
Schematic representation of IMR features in order of increasing sophistication/maturity. Formatting is a core requirement of multimedia reporting. Interactivity and structure both provide additional independent functionality

Any given IMR deployment may achieve some or all the above goals. Figure 7 proposes a schema for thinking about IMR features as levels of maturity. Enhanced formatting of the textual report is the foundation of IMR. These reports exist on a wide spectrum of interactivity and structure characteristics. For example, a PDF report with hyperlinks to an image viewer may have formatting and basic interactivity but lack a coded structure. An IMR encoded as an FHIR DiagnosticReport, as proposed in the IHE IMR Technical Supplement [20], enables the report to have a coded structure. Interactivity and formatting are mediated by the text of the report encoded as HTML.

## Conclusion

Increasing utilization of IMR requires standardization between image repositories, report authoring tools, and EHRs. The outlined workflow has highlighted the need for standardization in report authoring, report exchange, and report viewing. The IHE IMR Profile currently being developed will define the use of emerging standards for IMR exchange and display. The IHE process invites public comments on the proposed profile and facilitates a Connectathon for solution developers to test their applications. Further work is needed to define vendor neutral orchestration between report authoring and image display actors. Vendors must support emerging standards and provide IMR solution developers access without prohibitive costs. The creation of IMR requires certain prerequisites in image storage, annotation, and reporting systems. These prerequisites have been met by many radiology departments. Solution developers in other image-centric specialties including pathology, endoscopy, ophthalmology, and dermatology must adopt similar standards to enable interoperable, vendor neutral interactive multimedia reporting.

## References

[1] Hall FM (2009). The radiology report of the future. Radiology.

[2] Dunnick NR, Langlotz CP (2008). The radiology report of the future: a summary of the 2007 Intersociety Conference. J Am Coll Radiol.

[3] Nayak L, Beaulieu CF, Rubin DL, Lipson JA (2013). A picture is worth a thousand words: needs assessment for multimedia radiology reports in a large tertiary care medical center. Acad Radiol.

[4] Beesley SD, Patrie JT, Gaskin CM (2019). Radiologist adoption of interactive multimedia reporting technology. J Am Coll Radiol.

[5] Folio LR, Machado LB, Dwyer AJ (2018). Multimedia-enhanced radiology reports: concept, components, and challenges. Radiographics.

[6] Patel BN, Lopez JM, Jiang BG, Roth CJ, Nelson RC (2017). Image-rich radiology reports: a value-based model to improve clinical workflow. J Am Coll Radiol.

[7] Sadigh G, Hertweck T, Kao C (2015). Traditional text-only versus multimedia-enhanced radiology reporting: referring physicians’ perceptions of value. J Am Coll Radiol.

[8] Society of Imaging Informatics in Medicine: Interactive Multimedia Reporting, a HIMSS-SIIM Community Workgroup. https://siim.org/page/himss_wg_multimedia_interactive. Accessed 18 Dec 2021

[9] Roth CJ, Clunie DA, Vining DJ, et al.: Multispecialty enterprise imaging workgroup consensus on interactive multimedia reporting current state and road to the future: HIMSS-SIIM Collaborative White Paper. *J Digit Imaging. *34:495-522, 2021 10.1007/s10278-021-00450-5 PMC8329131 34131793

[10] Folio LR, Nelson CJ, Benjamin M, Ran A, Engelhard G, Bluemke DA (2015). Quantitative radiology reporting in oncology: survey of oncologists and radiologists. American Journal of Roentgenology.

[11] Rosenkrantz AB, Lui YW, Prithiani CP (2014). Development and enterprise-wide clinical implementation of an enhanced multimedia radiology reporting system. J Am Coll Radiol.

[12] Bellon E, van Cleynenbreugel J, Suetens P (1994). Multimedia e-mail systems for computer-assisted radiological communication. Med Inform.

[13] Schramm C, Goldberg M, Pagurek B (1989). Multimedia radiological reports: creation and playback. J Digit Imaging.

[14] Rowberg AH, Price TD: The need and user requirements for integrating images with radiology reports. In: *Proceedings from the Annual Symposium on Computer Applications in Medical Care* 163–167, 1991 PMC2247516 1807579

[15] Lowe HJ, Antipov I, Walker WK, Polonkey SE, Naus GJ: WebReport: a world wide web based clinical multimedia reporting system. In: *American Medical Informatics Association Annual Fall Symposium* 314–318, 1996 PMC2233165 8947679

[16] Kurdziel KA, Hopper KD, Zaidel M, Zukoski MJ (1996). Robo-Rad: an inexpensive user-friendly multimedia report system for radiology. Telemed J.

[17] Maloney K, Hamlet CT (1999). The clinical display of radiologic information as an interactive multimedia report. J Digit Imaging.

[18] Willemink MJ, Koszek WA, Hardell C (2020). Preparing medical imaging data for machine learning. Radiology.

[19] Do HM, Spear LG, Nikpanah M (2020). Augmented radiologist workflow improves report value and saves time: a potential model for implementation of artificial intelligence. Acad Radiol.

[20] IHE IMR Technical Supplement for Trial Implementation ver.1.0.0. Available at https://profiles.ihe.net/RAD/IMR/index.html. Accessed 13 May 2022

[21] Holder J, Tocino I, Facchini D (2021). Current state of radiology report release in electronic patient portals. Clin Imaging.

[22] Ellenbogen AL, Patrie JT, Gaskin CM (2021). Improving patient access to medical images by integrating an imaging portal with the electronic health record patient portal. J Am Coll Radiol.

[23] Cram D, Roth CJ, Towbin AJ (2016). Orders- versus encounters-based image capture: implications pre- and post-procedure workflow, technical and build capabilities, resulting, analytics and revenue capture: HIMSS-SIIM Collaborative White Paper. J Digit Imaging.

[24] Synoptic Reporting of Thyroid Nodules using TIRADS Enabled by Radiographer Documentation in RIS: Available at https://siim.org/page/20m_p_synoptic_reporting. Accessed 18 Dec 2021

[25] Radiology Technical Committee: IHE Radiology Technical Framework Supplement - AI Results. Available at https://www.ihe.net/uploadedFiles/Documents/Radiology/IHE_RAD_Suppl_AIR.pdf. Accessed 18 Dec 2021

[26] DICOM Standards Committee: DICOM PS3.4 2022b - Service Class Specifications. Available at https://dicom.nema.org/medical/dicom/current/output/html/part04.html. Accessed 18 Dec 2021

[27] Lee AY, Campbell JP, Hwang TS, Lum F, Chew EY (2021). American Academy of Ophthalmology: recommendations for standardization of images in ophthalmology. Ophthalmology.

[28] DICOM Standards Committee: DICOM PS3.6 2022b - Data Dictionary. Available at https://dicom.nema.org/medical/dicom/current/output/html/part06.html. Accessed 18 Dec 2021

[29] DICOM Standards Committee: DICOM PS3.18 2022b - Web Services. Available at https://dicom.nema.org/medical/dicom/current/output/chtml/part18/PS3.18.html. Accessed 18 Dec 2021

[30] Health Level 7 International: ImagingStudy - FHIR v4.0.1. Available at https://www.hl7.org/fhir/imagingstudy.html. Accessed 18 Dec 2021

[31] Healthcare Information and Management Systems Society: Digital Imaging Adoption Model (DIAM). Available at https://www.himss.org/what-we-do-solutions/digital-health-transformation/maturity-models/digital-imaging-adoption-model-diam. Accessed 18 Dec 2021

[32] Williams BJ, Bottoms D, Clark D, Treanor D (2019). future-proofing pathology part 2: building a business case for digital pathology. J Clin Pathol.

[33] Clunie D: Standardizing AI Annotations The DICOM Way. Presented at: SIIM Conference on Machine Intelligence in Medical Imaging; September 9–10 2018; San Francisco. Available at https://cdn.ymaws.com/siim.org/resource/resmgr/mimi18/presentations/18cmimi_ml-clunie.pdf. Accessed 18 Dec 2021

[34] Mongkolwat P, Kleper V, Talbot S, Rubin D (2014). The National Cancer Informatics Program (NCIP) Annotation and Image Markup (AIM) Foundation model. J Digit Imaging.

[35] PaLM Technical Committee: Pathology and Laboratory Medicine Technical Framework Supplement. Available at https://www.ihe.net/uploadedFiles/Documents/PaLM/IHE_PaLM_Suppl_RPC.pdf. Accessed 18 Dec 2021

[36] Vining, D, Tsimberidou, A, Garg, N, Markey, M, Ganapathi, T, Wang, J, Rosu, R, Jurca, M, Aghenitei, I: A Vision for Radiology Structured Reporting. Radiological Society of North America 2011 Scientific Assembly and Annual Meeting, November 26 - December 2, 2011, Chicago IL. http://archive.rsna.org/2011/11034318.html. Accessed 9 Jun 2022

[37] Goldberg-Stein S, Chernyak V (2019). Adding value in radiology reporting. J Am Coll Radiol.

[38] Beesley SD, Gaskin CM (2018). Interactive multimedia reporting: key features and experience in clinical practice. J Am Coll Radiol.

[39] Health Level 7 International: FHIRcast. Available at https://fhircast.org/. Accessed 18 Dec 2021

[40] World Wide Web Consortium: WebSub. Available at https://www.w3.org/TR/websub/. Accessed 21 Dec 2021

[41] Health Level 7 International: HL7 Context Management Specification (CCOW), Version 1.6. Available at https://www.hl7.org/implement/standards/product_brief.cfm?product_id=1. Accessed 19 Dec 2021

[42] Health Level 7 International: SMART Web Messaging Implementation Guide. Available at http://hl7.org/fhir/uv/smart-web-messaging/2020Sep/. Accessed 18 Dec 2021

[43] Srigley JR, McGowan T, Maclean A (2009). Standardized synoptic cancer pathology reporting: a population-based approach. J Surg Oncol.

[44] Leslie KO, Rosai J (1994). Standardization of the surgical pathology report: formats, templates, and synoptic reports. Semin Diagn Pathol.

[45] Rubin DL, Kahn CE (2017). Common Data Elements in Radiology. Radiology.

[46] Health Level 7 International: HL7 Version 2 Product Suite. Available at http://www.hl7.org/implement/standards/product_brief.cfm?product_id=185. Accessed 18 Dec 2021

[47] Jones S, Mazuryk J, Havener L, eds.: *Standards for Cancer Registries Volume V: Pathology Laboratory Electronic Reporting, Version 5*. North American Association of Central Cancer Registries, Inc.; 2020

[48] Dolin RH, Alschuler L, Beebe C (2001). The HL7 clinical document architecture. J Am Med Inform Assoc.

[49] Mehan WA, Brink JA, Hirsch JA (2021). 21st century cures act: patient-facing implications of information blocking. J Am Coll Radiol.

[50] IHE Radiology Technical Committee: IHE Radiology Technical Framework Supplement - Invoke Image Display. Available at https://wiki.ihe.net/index.php/Invoke_Image_Display. Accessed 18 Dec 2021

